# Supplementary material for: Patterns of Intron Gain and Loss in Fungi
Source: PLoS Biol. 2004 Nov 30;2(12):e422. doi: 10.1371/journal.pbio.0020422 (PMC532390; doi:10.1371/journal.pbio.0020422)
Supplement: Table S1 — Also available at http://genes.mit.edu/NielsenEtAl/. (4.3 MB ZIP). [file pbio.0020422.st001.zip › NielsenEtAl/html/1269.html]

AN4443.1.NCU06512.1.MG06712.1.FG10825.1


```
 CLUSTAL W (1.82) Multiple Sequence Alignments - Introns Inserted


Sequence 1: MG06712.1	766 aa
Sequence 2: FG10825.1	766 aa
Sequence 3: AN4443.1	763 aa
Sequence 4: NCU06512.1	769 aa
Alignment Length: 776 aa
Number Identitical Residues: 508 aa
Alignment Score (without introns) 22884


MG06712.1 	MVQSAILGFP~RMGVNRDLKKATEAY1WAGKISQDDLLAEAKRLRLAHWKIQKDAGVDII
NCU06512.1	MVQSSVLGFP2RMGVLRDLKKANEAY~WADKISQEALLAEGKRLRLAHWKIQKDAGVDII
FG10825.1 	MVQSAILGFP~RMGVNRDLKKATEAY1WGGKISQSDLLAEAKRLRLAHWKIQQDAGVDII
AN4443.1  	----------~-MGKLRDLKKATEAY~WGEKISRDELLSEGKRLRAEHWKIQKDAGVDII
          	            **  ******.*** *. ***:. **:*.****  *****:*******

MG06712.1 	PSNDFALYDQVLSHIQDFG0AVPERYSSSKLNPVDEYFAMGRGHQK---DG---VDVPSL
NCU06512.1	PSNDFAHYDHVLDHIQLFN0AVPERYTSQKLSPLDEYFAMGRGHQK---GG---VDVPAL
FG10825.1 	PSNDFALYDHVLHQIQDFG0AVPERYTKDGLDPIDQYFAMGRGHQK---EG---VDVPSL
AN4443.1  	PSNDFAFYDQVLDHIQLFG~VVPERYSKYNLHPLDEYFAMGRGLQKPAKDGQPAIDVPSL
          	****** **:** :** *. .*****:.  * *:*:******* **.:. *..::***:*

MG06712.1 	EMVKWFDSNYHYVKPTLQDNQTFTLTANPKAVNEFNEAKEAGINTRPVLVGPVSFLHLAK
NCU06512.1	EMVKWFDSNYHYVKPTLQDNQTFSLAKDPKPVREFLEAKEAGFQTRPVLVGPVSFLALGK
FG10825.1 	EMVKWFDSNYHYVKPTLQDGQTFKLAESPKAVADFKEAKDAGISTRPVLVGPVSFLHLGK
AN4443.1  	EMVKWFDSNYHYVKPTLQDNQTFKLAANPKPVVEFLEAKEAGIVTRPVILGPVSFLTLAK
          	*******************.***.*: .**.* :* ***:**: ****::****** *.*

MG06712.1 	ADRGQSVDPIDLLDKLVPVYEELLAKLKAAGAETVQIDEPVLVFDLPAKVKAAFKPTYEK
NCU06512.1	ADRGSSVDPITLLDKLVPVYVELLKQLKAAGAESVQIDEPVLVFDLRPEVKAAFKPAYEA
FG10825.1 	ADRDQKVDPIDLLEKLLPVYEQLLTQLKEAGAETVQIDEPVLVFDLPQKTKDAFKPAYEK
AN4443.1  	ADRGQTLDPISKIDELLPLYVELLTKLKEAGVEDVQIDEPVLVFDLPLKSKNAFKPAYEK
          	***...:***  :::*:*:* :** :** **.* ************  : * ****:** 

MG06712.1 	FASLGDKIPKLVFATYFGDIVHNLDLVP--KDVYAVHVDLVRNPEQLETVVGALGPKTIL
NCU06512.1	IAAAGDAVPKVVVATYFGDIVHNFDVLPAFSGAAGLHVDLVRNPEQLEPVLKQLGPNQIL
FG10825.1 	FASLGDKIPKVVFTTYFGDIVHNLDLLP--KDVYGLHIDLVRNPEQLDKVLGAIGSNTIL
AN4443.1  	LGSLGAQAPRLVLATYFGDIVHNIDVLPALHNIYGIHIDLVRNPEQLDSVIGALGPKQVL
          	:.: *   *::*.:*********:*::*:  .  .:*:*********: *:  :*.: :*

MG06712.1 	SAGIVDGRNIWKTNFQKAIETVESAIQKLGKERVIVATSSSLLHTPHTLASEKKLDPEIA
NCU06512.1	SAGVVDGRNIWKNDFAKSLEILQTAVKALGSERVIVATSSSLIHTPHTLASEKKLPSDVY
FG10825.1 	SAGVVDGRNIWKTNLKRAIEIVETAVQKLGKDRVIAATSSSLLHTPHTLASEKKLDTEIA
AN4443.1  	SAGVVDGRNIWKTNFKAAIEKVELAIQKLGKDRVIVSTSSSLLHVPHTLASEKNLDPEVQ
          	***:********.::  ::* :: *:: **.:***.:*****:*.********:* .:: 

MG06712.1 	DWFSFASEKAVEVAIIAKAVTEGPAAVREQLEANAKSMNARATSSRTNDPKVKERQSKIV
NCU06512.1	EWFSFAVEKVKEVATLAKAVTE-PEAVKAELEANAAAIKARTDSKRTNDPAVKERQAQVT
FG10825.1 	DWFSFATEKASEIALIAKAVTEGPATVREQLEANAKSIKARADSPRTNDPQVKDRQSKVT
AN4443.1  	DWFSFAVEKTSEVVVIAKAVTEGPAAVREQLEANAKSVQARASSKRTNDPKVKERQAAVT
          	:***** **. *:. :******.* :*: :***** :::**: * ***** **:**: :.

MG06712.1 	ESDYNRKSEFPTRISQQQAKLNLPLFPTTTIGSFPQTQTIRAQRAKLTKKEIDAEQYAKF
NCU06512.1	PEQHNRKAPFNTRYAEQKKHLSLPLFPTTTIGSFPQTSEIRVQRNKFTKGEISAEEYERF
FG10825.1 	QKDYERKSEFTTRISQQQKKLNLPLFPTTTIGSFPQTKEIRIQRNKFTKGEITVEEYDRF
AN4443.1  	PEQHNRKSPFPVRIAEQTKSIKLPLFPTTTIGSFPQTKEIRIQRNKFTKGEITAEEYEKF
          	 .:::**: * .* ::*   :.***************. ** ** *:** ** .*:* :*

MG06712.1 	IEEEIENNVKIQEELGLDVFVHGEPERNDMVQFFGERLDGYAFTTHAWVQSYGSRCVRPP
NCU06512.1	IEKEIELAVKIQDELDLDVYVHGEPERNDMVQYFGERLNGYVFTTHAWVQSYGSRCVRPP
FG10825.1 	IEKEIEENVKIQEELDLDVLVHGEPERNDMVQFFGERFQGYAFTTHAWVQSYGSRCVRPP
AN4443.1  	IEKEIAEVVKIQEELGLDVLVHGEPERNDMVQYFGERLTGYVFTTHAWVQSYGSRCVRPP
          	**:**   ****:**.*** ************:****: **.******************

MG06712.1 	IIVGDISRPAPMTVKESRYAVEISKKPMKGMLTGPVTCLRWSFPRDDVHQSVQAEQLALA
NCU06512.1	IIVGDISRPAPMTVKESKYAASISKKPMKGMLTGPVTCLRWSFPRVDVHQSVQCQQLALA
FG10825.1 	IIVGDISRPNPMTVKESKYAVSVSKKPMKGMLTGPVTCLRWSFPRDDVHQSVQAEQLALA
AN4443.1  	IIVGDISRPAPMTVKESKYAVSISSKPMKGMLTGPITCLRWSFPRDDVHQSVQAQQLALA
          	********* *******:**..:*.**********:********* *******.:*****

MG06712.1 	LRDEVVDLEKAGVDVIQVDEPALREGLPLRSGKERDAYLQWAVKAFKLSTCGVEDSTQIH
NCU06512.1	LRDEVVDLEKNGIYVIQVDEPALREGLPLRKGQEREAYLKWAVDSFKLATAGVENSTQIH
FG10825.1 	LRDEVVDLEKAGIDVIQVDEPALREGLPLRSGEERDAYLKWAVQAFRLSTAGVEDATQIH
AN4443.1  	LRDEVVDLEAAGVKVIQVDEPALREGLPLRAGKEREDYLQWAVAAFRLSTSGVSDGTQIH
          	*********  *: **************** *:**: **:*** :*:*:*.**.:.****

MG06712.1 	SHFCYSEFQDFFHAIAALDADVLSIENSKSDAKLLKVFVDSAYPRHIGPGVYDIHSPRVP
NCU06512.1	SHFCYSEFQDFFHAIAALDADVLSIENSKSDAKLLKVFIDEEYPRHIGPGVYDIHSPRVP
FG10825.1 	SHFCYSEFQDFFHAIAALDADVLSIENSKSDAKLLRVFVDSEYPRHIGPGVYDIHSPRVP
AN4443.1  	SHFCYSEFQDFFHAIAALDADVLSIENSKSDAKLLKVFIDEAYPRHIGPGVYDIHSPRVP
          	***********************************:**:*. ******************

MG06712.1 	SEQEIKDRIEEMLQYLKPEQLWIDPDCGLKTRQWKETKEALTNMVNAAKYFRAKYAK--
NCU06512.1	TLEEFKQRIEEMLAYLKPEQLWINPDCGLKTRKWDEVKGALSHMVEAAKYFREKYANKA
FG10825.1 	SEQEIKDRIEEMLQFLKPEQLWIDPDCGLKTRQWKETKEALTNMVNAAKYFRNKYAK--
AN4443.1  	SEQEIKDRVEEMLAYLRPEQLWINPDCGLKTRQWPETKAALSNLVQAAKYFREKYAK--
          	: :*:*:*:**** :*:******:********:* *.* **:::*:****** ***:
```
